# Does obesity affect the diagnostic value of various biomarkers for periprosthetic joint infections? A single center retrospective study

**DOI:** 10.3389/fcimb.2025.1483503

**Published:** 2025-02-18

**Authors:** Wei-peng Shi, Ya-ping Jiang, Ting-yu Wu, Ying-zhen Wang, Ying-ze Zhang, Tao Li

**Affiliations:** ^1^ Department of Orthopaedic Surgery, The Affiliated Hospital of Qingdao University, Qingdao, China; ^2^ Department of Oral Implantology, The Affiliated Hospital of Qingdao University, Qingdao, China; ^3^ Department of Orthopaedics, The Third Hospital of Hebei Medical University, Shijiazhuang, China

**Keywords:** obesity, periprosthetic joint infection, CRP, CAR, ESR

## Abstract

**Objective:**

The aim of this study was to examine the impact of obesity on the diagnosis of periprosthetic joint infections (PJI) by assessing the levels and diagnostic efficacy of biomarkers.

**Methods:**

A total of 254 patients were divided into obese group (n=59) and non-obese group (n=195) according to BMI. Each group was further divided into the PJI group and the AF group. Data on CRP, ESR, fibrinogen, D-dimer, CRP-albumin ratio (CAR), CRP-lymphocyte ratio (CLR), and CRP-monocyte ratio (CMR) were collected from all patients. ROC curve was performed to evaluate the diagnostic values of these biomarkers.

**Results:**

The levels of biomarkers were significantly higher in PJI patients compared to the AF patients in both the obese and non-obese groups (P < 0.001), but the levels of biomarkers were similar between the obese and non-obese groups. In the obese group, CRP exhibited the highest diagnostic value (AUC=0.982). In the non-obese group, CAR demonstrated the highest diagnostic value (AUC =0.935). Subgroup analysis showed no significant differences in biomarker levels (P > 0.05).

**Conclusions:**

Obesity did not affect biomarker levels in patients with PJI. But for obese patients, the diagnostic thresholds for CRP and ESR are higher, and clinical diagnosis should be careful to avoid false positives. CRP and CAR were identified as the most effective biomarkers for diagnosing PJI in the obese and non-obese groups, respectively.

## Introduction

With the global population increasing and population aging progressing, the number of total joint arthroplasties (TJAs) is expected to surpass 4 million per year in the UK and the United States by 2030 (Kapadia et al., 2016). TJA is a common treatment for end-stage osteoarthritis, effectively reducing pain and promoting joint function recovery. However, periprosthetic joint infection (PJI), although rare with an incidence of only 0.5-2% (Whitehouse et al., 2016; Gundtoft et al., 2017; Koh et al., 2017), poses a severe and devastating complication following TJA, imposing significant burdens on patients and society both in terms of health and economic impact. The incidence of PJI is projected to rise due to the increasing prevalence of obesity, diabetes, and other comorbidities (Kapadia et al., 2016). Numerous studies have already demonstrated that obesity/morbid obesity is a significant risk factor for TJA failure (Houdek et al., 2015; Watts et al., 2015; Kapadia et al., 2016; Roth et al., 2019).

Since the 1980s, the body mass index (BMI) and obesity rates among the Chinese population have been steadily increasing (Wang et al., 2021). Obesity not only serves as a significant risk factor for cardiovascular and kidney diseases (Wang et al., 2021) but also impacts the levels of various serum inflammatory biomarkers (Cohen et al., 2021). Adipose cells play a role in synthesizing the pro-inflammatory cytokine IL-6, which subsequently leads to increased production of C-reactive protein (CRP) by the liver (Laaksonen et al., 2004; Cohen et al., 2021). Obese individuals have been found to exhibit higher levels of fibrinogen compared to those with normal body weight, with an increase of 93.5 mg/dl (95% CI, 72.9-114.1) based on reference values from individuals with normal body weight (Nguyen et al., 2009). Leff et al. have discovered that obese patients who exceed their ideal weight by 45 kg exhibit significantly higher average erythrocyte sedimentation rates (ESR) compared to patients with normal weight (17 mm/h vs. 4 mm/h, P=0.015) (Leff and Akre, 1986). PJI and aseptic failure (AF) share certain clinical symptoms, such as pain and joint swelling. Therefore, accurately distinguishing between PJI and AF holds crucial importance for guiding targeted treatments in the future. PET-CT is an important tool for diagnosing PJI, and whole-body PET/CT may detect other infectious lesions, which can have an impact on subsequent treatment strategies (Roschke et al., 2021). A study reported abnormalities in 99mTC labeled scintigraphy in patients with early prosthetic infections, but the study did not discuss the low specificity of the test (Matthews et al., 2009). However, these technological methods are expensive and difficult to widely use in primary hospitals. There is currently a lack of research regarding the impact of obesity on the levels of various biomarkers and the diagnostic capability for PJI patients. Hence, our study aimed to incorporate the CAR, CLR, and the newly introduced CMR alongside traditional biomarkers (CRP and ESR) and fibrinolytic biomarkers (fibrinogen and D-dimer), all of which have demonstrated promising diagnostic value in previous studies conducted by our research team (Shi et al., 2022; Shi et al., 2023) and by others (Pevzner et al., 2011).

The primary research objectives of this study were as follows: (1) to investigate the impact of obesity on the levels of serum inflammatory biomarkers in PJI patients; (2) to determine the optimal diagnostic biomarkers for PJI patients in both the obese and non-obese groups; (3) to explore the possibility of identifying an ideal biomarker that could be universally applied to both groups of patients; (4) and to assess the diagnostic value of various biomarkers in subgroup analyses of obese and non-obese patients.

By addressing these objectives, we aimed to enhance our understanding of the relationship between obesity and serum inflammatory biomarkers in PJI patients, identify effective diagnostic biomarkers tailored to the specific needs of obese and non-obese patients, and evaluate the diagnostic performance of these biomarkers within subgroups.

## Methods

### Study population

This single-center retrospective study was conducted in accordance with the principles outlined in the Declaration of Helsinki. Approval for the study was obtained from the Institutional Review Board (IRB) of the Affiliated Hospital of Qingdao University. Data were collected from the electronic case system of patients who underwent total knee or hip revision surgery at our institution between June 2013 and April 2023. To ensure accuracy, certain exclusion criteria were applied. Patients with periprosthetic fractures and dislocation were initially excluded. Additionally, patients with the following conditions were excluded from the study: (1) malignant tumors; (2) hematological diseases (thrombocytopenia, thrombosis, etc.); (3) autoimmune diseases (rheumatoid arthritis, systemic lupus erythematosus, ankylosing spondylitis, etc.); (4) infections in other body parts; (5) recent use of anticoagulants or antibiotics; and (6) missing data. After the screening process, a total of 254 patients were included in the final analysis. Considering that all patients were of Chinese descent, those with a BMI greater than 28 kg/m^2^ were classified as obese, while the remaining patients were classified as non-obese. Following the criteria set forth by the 2018 International Consensus Conference (ICM) (Parvizi et al., 2018), each patient group was further divided into the PJI group and the AF group.

### Data extraction

The baseline data for all the included patients primarily consisted of age, gender, height, weight, and the affected joints. On the day of admission or early morning of the following day, a specialist nurse collected fasting venous blood samples from the patients, which were then promptly sent to the laboratory for testing within 1 hour. The collected blood samples were used to measure serum levels of CRP, ESR, neutrophils, lymphocytes, monocytes, and albumin. Additionally, relevant biomarker ratios were calculated based on these measurements. Moreover, in order to further improve the accuracy of diagnosis, during the surgical procedure, synovial fluid or pus samples were obtained from patients who were either suspected or confirmed to have PJI. These samples were subjected to culture under both aerobic and anaerobic conditions. Concurrently, histopathological examinations were conducted on the tissues or bones surrounding the prosthesis.

### Statistical analyses

All statistical analyses in this study were performed using SPSS version 26.0 (IBM Inc., Armonk, NY, USA). Continuous variables were presented as mean ± standard deviation (SD), while categorical variables were expressed as percentages (%). Student’s t-test was employed to compare normally distributed continuous variables between two groups, whereas the Mann-Whitney U test was used for non-normally distributed continuous variables. The chi-square test was applied to analyze categorical variables. Statistical significance was defined as P<0.05. Receiver operating characteristic (ROC) curves were constructed to calculate the area under the curve (AUC) for each biomarker, determining their diagnostic value. The 95% confidence intervals (CI), sensitivity, specificity, positive predictive value (PPV), and negative predictive value (NPV) were also calculated based on the Youden index. The diagnostic value of each biomarker was categorized into five levels based on the AUC: excellent (0.900-1.000), good (0.800-0.899), fair (0.700-0.799), poor (0.600-0.699), and no diagnostic ability (0.500-0.599). All figures were generated using GraphPad Prism 8.0.2 (GraphPad Software Inc., San Diego, CA, USA).

## Results

### Demographic characteristics of obese and non-obese patients

A total of 254 patients were included in the study, comprising 59 obese patients and 195 non-obese patients. The results indicated that the obese group had a higher proportion of PJI patients compared to the non-obese group, suggesting that obesity was indeed a risk factor for PJI. There were no significant differences in age and gender distribution between the PJI and AF groups, regardless of whether patients were obese or non-obese (P>0.05). In the obese group, a statistically higher proportion of patients with knee infections (p=0.023), whereas in the non-obese group, the proportions of knee and hip joint infections were similar (**Table 1**).

**Table 1:** Demographic characteristics of obesity and non-obesity patients.

	Obese		P value	Non-obese		P value
	PJI (n=30)	AF (n=29)		PJI (n=61)	AF (n=134)	
Age(y)	64.40 ± 9.85	65.34 ± 8.12	0.689	64.98 ± 8.62	65.32 ± 9.77	0.825
Gender (female, %)	16 (53.33)	17 (58.62)	0.683	31 (50.82)	70 (52.24)	0.854
Joint			<0.001			<0.001
Knee	24	8		27	27	
Hip	6	29		34	107	

PJI, periprosthetic joint infections, AF, aseptic failure.

### Comparison of the levels of biomarkers in the obese and non-obese patients

**Figure 1:**
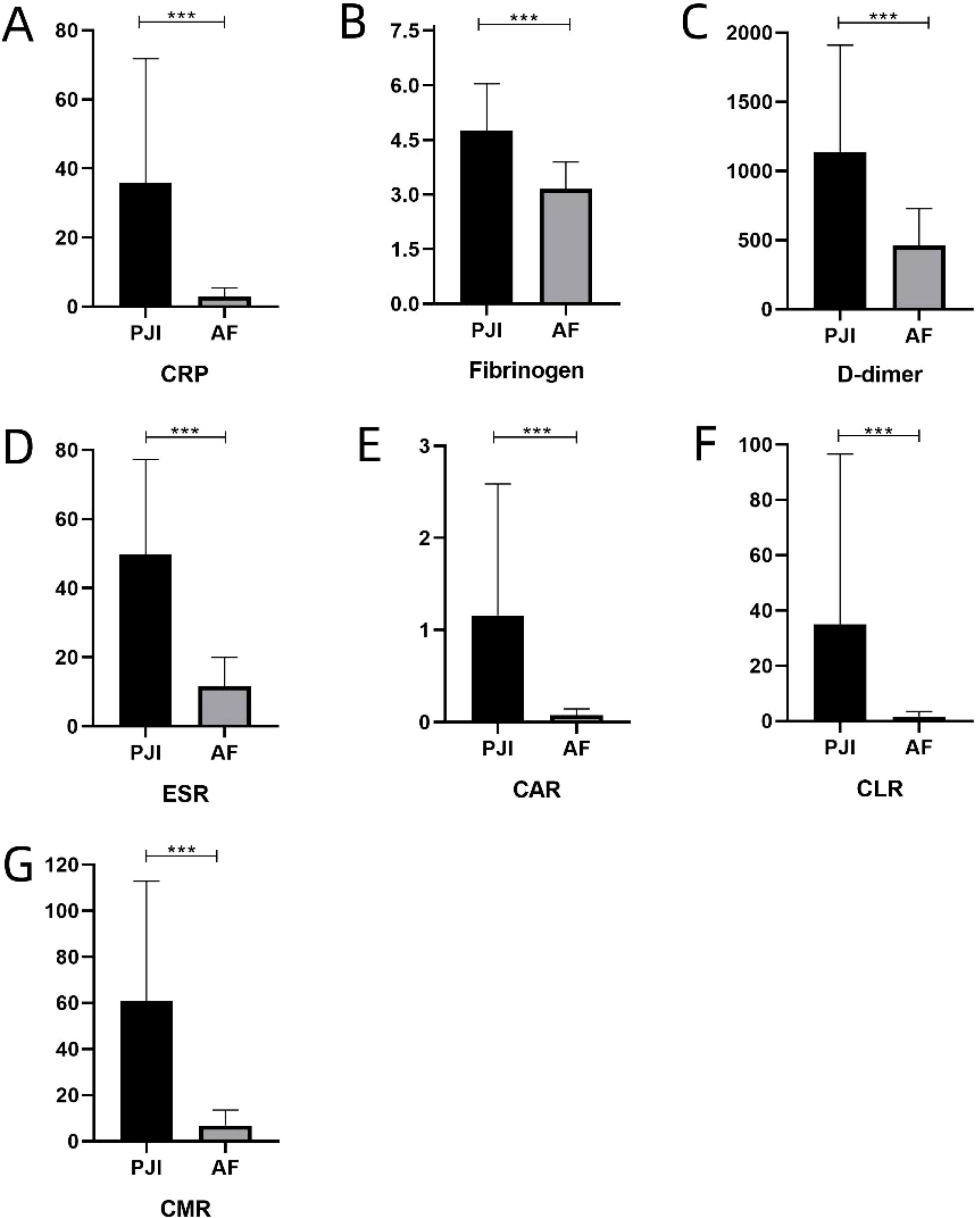
**(A-G)** represents the levels of various serum inflammatory biomarkers in obese patients, respectively. C-reactive protein, ESR, erythrocyte sedimentation rate, CAR, CRP-albumin ratio, CLR, CRP-lymphocyte ratio, CMR, CRP-monocyte ratio. ***: P<0.001.

**Figure 2:**
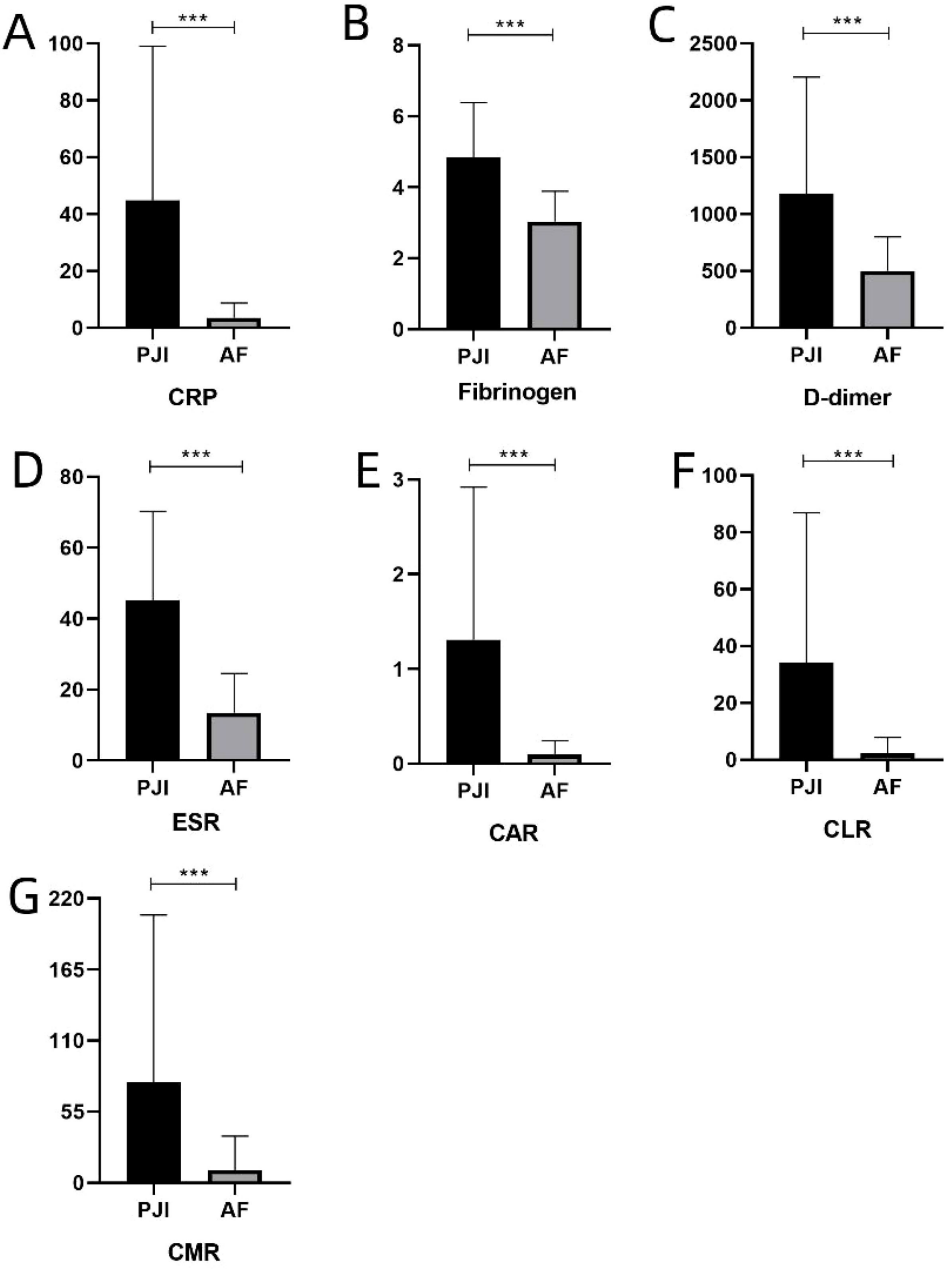
**(A-G)** represents the levels of various serum inflammatory biomarkers in non-obese patients, respectively. C-reactive protein, ESR, erythrocyte sedimentation rate, CAR, CRP-albumin ratio, CLR, CRP-lymphocyte ratio, CMR, CRP-monocyte ratio. ***: P<0.001.

Compared with the AF group, the PJI group showed significantly elevated serum levels and ratios of various biomarkers (CAR, CLR, and CMR) in both the obese and non-obese groups (P<0.001). Detailed results can be found in **Table 2** and **Figures 1**, **2**.

**Table 2:** Comparison of the levels of biomarkers in the obesity and non-obesity patients.

	Obese		P value	Non-obese		P value
	PJI (n=30)	AF (n=29)		PJI (n=61)	AF (n=134)	
CRP (mg/L)	35.98 ± 35.80	2.95 ± 2.49	<0.001	44.91 ± 54.08	3.54 ± 5.15	<0.001
Fibrinogen(g/L)	4.75 ± 1.29	3.16 ± 0.73	<0.001	4.83 ± 1.55	3.04 ± 0.85	<0.001
D-dimer(ng/mL)	1137.59 ± 771.08	461.43 ± 266.77	<0.001	1177.64 ± 1025.90	500.12 ± 299.30	<0.001
ESR (mm/h)	49.84 ± 27.46	11.57 ± 8.42	<0.001	45.20 ± 25.07	13.52 ± 11.06	<0.001
CAR	1.16 ± 1.43	0.07 ± 0.07	<0.001	1.31 ± 1.61	0.09 ± 0.15	<0.001
CLR	35.04 ± 61.47	1.74 ± 1.76	<0.001	34.10 ± 52.81	2.32 ± 5.53	<0.001
CMR	60.72 ± 52.11	6.89 ± 6.71	<0.001	77.61 ± 129.47	9.78 ± 26.25	<0.001

CRP, C-reactive protein, ESR, erythrocyte sedimentation rate, CAR, CRP-albumin ratio, CLR, CRP-lymphocyte ratio, CMR, CRP-monocyte ratio.

**Figure 3:**
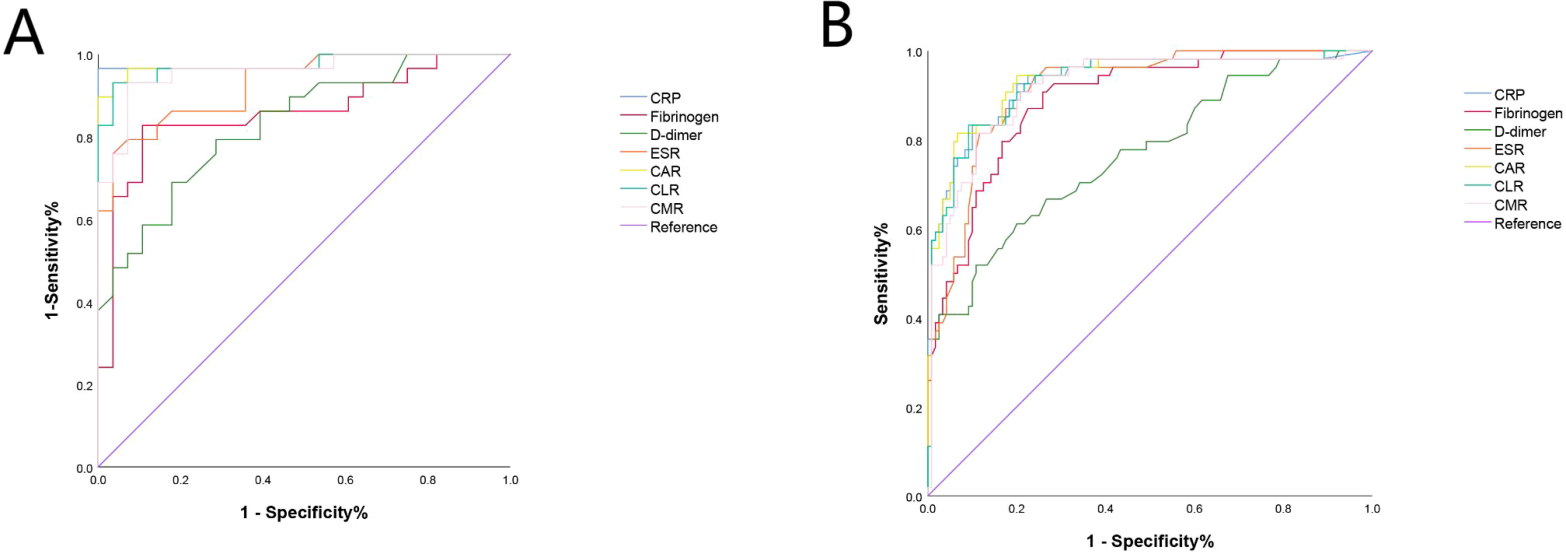
The ROC of CRP, Fibrinogen, D-dimer, ESR, CAR, CLR and CMR in obese and non-obese patients. **(A)** In the obese group, the ROC analysis indicated that CRP had the highest diagnostic value for PJI with an AUC of 0.982, followed by CAR with an AUC of 0.978. **(B)** In the non-obese group, the ROC analysis indicated that CAR had the highest diagnostic value for PJI with an AUC of 0.935, followed by CRP with an AUC of 0.932. CRP, C-reactive protein, ESR, erythrocyte sedimentation rate, CAR, CRP-albumin ratio, CLR, CRP-lymphocyte ratio, CMR, CRP-monocyte ratio.

In the obese group, the AUC (95% CI) values for the seven biomarkers were as follows: CRP - 0.982 (0.945, 1.000), fibrinogen - 0.858 (0.755, 0.961), D-dimer - 0.830 (0.727, 0.934), ESR - 0.928 (0.865, 1.000), CAR - 0.978 (0.940, 1.000), CLR- 0.973 (0.933, 1.000), and CMR - 0.959 (0.911, 1.000). CRP exhibited the highest diagnostic value, with an optimal cutoff value of 8.38, a sensitivity of 96.6%, and a specificity of 100.0%. CAR had the second-highest diagnostic value, with an optimal cutoff value of 0.26, a sensitivity of 89.7%, and a specificity of 100.0%. Based on a further calculation using the Youden index, the PPV for the seven biomarkers or ratios was 100.0%, 85.7%, 80.0%, 95.3%, 100.0%, 96.6%, and 93.3%, respectively. The NPV was 96.7%, 80.7%, 71.9%, 80.0%, 90.6%, 93.3%, and 93.1%, respectively (**Table 3**; **Figure 3A**).

**Table 3:** The diagnostic value of biomarkers in the obese and non-obese PJI patients.

	Obese								
	AUC	95%CI	Youden index	Optimal cutoff value	Sensitivity (%)		Specificity (%)	PPV (%)	NPV (%)
CRP (mg/L)	0.982	(0.945,1.000)	0.966	8.38	96.6		100.0	100.0	96.7
Fibrinogen (g/L)	0.858	(0.755,0.961)	0.720	3.73	82.8		89.3	85.7	80.7
D-dimer (ng/mL)	0.830	(0.727,0.934)	0.511	690.00	69.0		82.1	80.0	71.9
ESR (mm/h)	0.928	(0.865,1.000)	0.723	29.00	75.9		96.4	95.3	80.0
CAR	0.978	(0.940,1.000)	0.897	0.26	89.7		100.0	100.0	90.6
CLR	0.973	(0.933,1.000)	0.895	5.46	93.1		96.4	96.6	93.3
CMR	0.959	(0.911,1.000)	0.860	17.63	93.1		92.9	93.3	93.1

Non-obese									
AUC		95%CI	Youden index	Optimal cutoff value	Sensitivity (%)	Specificity (%)		PPV (%)	NPV (%)
0.932		(0.889,0.975)	0.733	7.36	83.3	90.0		76.1	92.2
0.892		(0.843,0.940)	0.649	3.36	90.7	74.2		60.0	93.3
0.769		(0.690,0.847)	0.411	685.00	61.1	80.0		55.9	82.1
0.914		(0.872,0.956)	0.703	16.95	94.4	75.8		65.1	96.0
0.935		(0.894,0.977)	0.748	0.27	81.5	93.3		83.3	91.9

CRP, C-reactive protein, ESR, erythrocyte sedimentation rate, CAR, CRP-albumin ratio, CLR, CRP-lymphocyte ratio, CMR, CRP-monocyte ratio, PPV, positive predictive value, NPV, negative predictive value.

In the non-obese group, the ROC analysis indicated that CAR had the highest diagnostic value for PJI with an AUC of 0.935 (95% CI: 0.894, 0.977), followed by CRP with an AUC of 0.932 (95% CI: 0.889, 0.975). The subsequent biomarkers in terms of diagnostic value were CLR (AUC=0.929, 95% CI: 0.866, 0.973), CMR (AUC=0.918, 95% CI: 0.872, 0.965), ESR (AUC=0.914, 95% CI: 0.872, 0.956), fibrinogen (AUC=0.892, 95% CI: 0.843, 0.940), and D-dimer (AUC=0.769, 95% CI: 0.690, 0.847) (**Table 3**; **Figure 3B**).

Two important findings should be noted. Firstly, the optimal cutoff values of the two traditional biomarkers (CRP and ESR) were higher in the obese group compared to the non-obese group (8.38 vs. 7.36 for CRP; 29.00 vs. 16.95 for ESR). This finding suggested that obesity could affect the diagnostic thresholds for CRP and ESR in PJI. Secondly, D-dimer showed the lowest sensitivity and poor specificity in both the obese and non-obese groups.

### Comparison of biomarkers in the subgroups

The subgroup analysis based on culture results, affected joints, and acute/chronic infections aimed to investigate whether various biomarkers still had diagnostic value in different situations within the obesity and non-obesity groups. The results indicated that there were relative differences in the levels of various biomarkers in each subgroup, regardless of obesity status. However, these differences were not statistically significant (P>0.05), suggesting that the diagnostic value of the biomarkers might not vary significantly across different subgroups (**Table 4**).

**Table 4:** Comparison of biomarkers in the obese and non-obese PJI subgroups.

	Obese										
	Culture positive PJI (n=21)	Culture negative PJI (n=9)		P value	Acute PJI (n=3)	Chronic PJI (n=27)		P value	Knee PJI (n=24)	Hip PJI (n=6)	P value
CRP (mg/L)	35.81 ± 38.15	36.78 ± 31.74		0.967	24.27 ± 10.45	37.28 ± 37.46		0.196	35.81 ± 32.20	36.68 ± 51.60	0.970
Fibrinogen(g/L)	4.74 ± 1.05	4.79 ± 1.81		0.935	4.29 ± 0.60	4.80 ± 1.34		0.292	4.75 ± 1.18	4.76 ± 1.79	0.997
D-dimer(ng/mL)	1262.00 ± 846.11	861.11 ± 505.98		0.127	1553.33 ± 1938.10	1089.62 ± 585.04		0.719	1231.30 ± 823.74	778.33 ± 385.61	0.068
ESR (mm/h)	50.21 ± 27.48	49.00 ± 29.05		0.917	36.03 ± 12.22	51.38 ± 28.37		0.146	51.01 ± 26.74	45.18 ± 32.43	0.697
CAR	1.15 ± 1.55	1.17 ± 1.16		0.982	0.62 ± 0.31	1.21 ± 1.49		0.094	1.07 ± 1.06	1.51 ± 2.55	0.697
CLR	34.38 ± 68.70	36.60 ± 43.51		0.916	15.00 ± 10.91	37.27 ± 64.45		0.124	37.83 ± 66.61	23.90 ± 36.35	0.500
CMR	62.21 ± 57.00	57.24 ± 41.28		0.792	52.13 ± 29.70	61.67 ± 54.35		0.661	65.50 ± 55.44	41.59 ± 32.64	0.194

	Non-obese								
	Culture positive PJI(n=42)	Culture negative PJI(n=19)	P value	Acute PJI (n=8)	Chronic PJI (n=53)	P value	Knee PJI (n=27)	Hip PJI (n=34)	P value
	51.56 ± 57.96	30.23 ± 42.03	0.111	44.98 ± 53.53	44.90 ± 54.67	0.997	41.27 ± 55.62	47.45 ± 53.52	0.686
	4.93 ± 1.62	4.61 ± 1.39	0.428	5.09 ± 1.10	4.79 ± 1.61	0.523	4.65 ± 1.78	4.97 ± 1.35	0.448
	1129.21 ± 921.81	1285.88 ± 1252.62	0.648	1082.86 ± 962.56	1191.46 ± 1043.75	0.790	1400.00 ± 1325.37	1029.39 ± 752.75	0.243
	45.55 ± 26.41	44.44 ± 22.55	0.867	48.09 ± 23.88	44.75 ± 25.44	0.724	45.73 ± 26.58	44.76 ± 24.16	0.885

CRP, C-reactive protein, ESR, erythrocyte sedimentation rate, CAR, CRP-albumin ratio, CLR, CRP-lymphocyte ratio, CMR, CRP-monocyte ratio, PJI, periprosthetic joint infections, AF, aseptic failure.

## Discussion

We conducted a comprehensive comparison of the diagnostic value of traditional inflammatory biomarkers (CRP and ESR), fibrinolytic biomarkers (fibrinogen and D-dimer), and previously established biomarkers (CAR, CLR, and CMR) in PJI patients, considering their obesity status. Our study findings revealed that the levels of serum inflammatory markers in obese PJI patients did not show a significant increase compared to the AF group. Among all biomarkers, CRP and CAR emerged as the most effective indicators for diagnosing PJI in obese and non-obese patients, respectively. Notably, the optimal cutoff value for CRP was higher in the obese group than in the non-obese group, while no significant difference was observed in CAR between the two groups. These results highlighted the importance of considering patients’ BMI when using CRP as a diagnostic indicator for PJI to avoid false positive results. Furthermore, it is noteworthy that all biomarkers performed poorly in subgroup analyses of obese and non-obese patients, indicating limited diagnostic ability in these specific populations.

CRP, as a widely studied traditional inflammatory biomarker, is known to be an acute-phase protein synthesized by liver cells in response to inflammation triggered by various factors, such as infection or obesity. Recent research has highlighted the complex relationship between obesity and chronic inflammation, with studies such as demonstrating that elevated CRP levels are not only indicative of acute infections but also reflect chronic inflammatory states associated with obesity. These findings further underscore the importance of developing tailored diagnostic thresholds for obese patients (Marongiu et al., 2020). Previous studies have demonstrated a positive association between CRP concentration and increasing BMI. For instance, Nguyen et al. have observed that as BMI increases, CRP concentration rises from 0.11 ± 0.03 mg/dl to 0.73 ± 0.09 mg/dl, with the strongest association seen in individuals with a BMI > 40 kg/m^2^ (Nguyen et al., 2009). Similarly, Visser et al. have reported elevated odds ratios (OR) for elevated CRP in obese males (OR: 2.13, 95% CI: 1.56-2.91) and females (OR: 6.21, 95% CI: 4.94-7.81) (Visser et al., 1999). Cohen et al. have also demonstrated significantly increased OR for elevated CRP across all BMI groups compared to individuals with normal BMI, irrespective of gender (Houdek et al., 2015). Furthermore, ESR levels have been found to be elevated in obese patients (Houdek et al., 2015). Given this existing research, it would be reasonable to expect higher CRP and ESR levels in obese PJI patients compared to non-obese PJI patients.

Probasco and his colleagues found that elevated CRP and ESR levels in preoperative inflammation are positively correlated with BMI, and they believed that traditional biomarkers should be used with caution in the diagnosis of obesity PJI (Probasco et al., 2020). However, in our study, we did not observe a significant difference in CRP and ESR levels between the obese and non-obese groups. Several factors may contribute to these unexpected findings. Firstly, obesity is characterized by chronic inflammation, and while inflammatory biomarkers may increase in obese patients, PJI represents a more acute and severe form of inflammation that can have a greater impact on markers like CRP and ESR. Secondly, the sample size of obese PJI patients in our study was relatively small compared to previous studies, which may have introduced greater variability and potential errors. Future large-scale research is needed to confirm these conclusions regarding inflammation. Additionally, it is important to consider that BMI is influenced by race (Issa et al., 2014), and variations in grouping criteria can also lead to discrepancies in results.

While the levels of CRP and ESR in obese PJI patients were not found to be significantly higher than those in non-obese groups, this study identified higher optimal cutoff values for diagnosing PJI using CRP and ESR. Moreover, both sensitivity and specificity were higher compared to our previous studies (Shi et al., 2021). Although these results should be interpreted cautiously due to the limited sample size, they indicated that traditional biomarkers still held considerable diagnostic value in obese PJI patients. Currently, clinical practice does not differentiate between obese and non-obese PJI patients, and using lower cutoff values for CRP and ESR may result in the overtreatment of obese individuals. On the other hand, the CAR, which is previously discovered in our study, demonstrated good performance in both obese and non-obese PJI patients. Importantly, there was no significant difference in the optimal cutoff value between the two patient groups. Thus, we recommend considering the correlation between CRP and CAR to enhance the accuracy of PJI diagnosis.

A recent study has highlighted that hip and knee infections are distinct issues. Specifically, in obese and severely obese patients with hip infection, higher rates of polymicrobial infection and Enterococcus are observed, while severely obese patients show a higher incidence of Gram-negative infections. However, this phenomenon is not observed in knee infections (Lowik et al., 2019). The underlying reason may be attributed to the increase in bacterial colonization on the hip skin, particularly in the groin, as BMI rises (OR=1.15, 95% CI: 1.03-1.29, P=0.01) (Boni et al., 2018). Conversely, in our present study, we observed a higher proportion of knee infections in the obese group. We speculated that this could be due to the hip joint’s proximity to the perineum, leading to more meticulous disinfection by medical professionals. Additionally, the knee joint is more exposed and poses an increased risk of postoperative infection. Another contributing factor could be the small sample size and inherent data biases, which may have resulted in contradictory outcomes.

Furthermore, another study has proposed that obese PJI patients (BMI > 35 kg/m^2^) exhibit a higher rate of positive intraoperative cultures, likely due to challenges in disinfection and lower tissue concentration of cefazolin in obese individuals (Font-Vizcarra et al., 2011; Pevzner et al., 2011). In our present study, the culture-positive rate in the obese group was 70.0% (21/30), while it was 68.9% (42/61) in the non-obese group. However, it is important to note that our study was retrospective and unable to accurately determine whether each patient received antibiotic treatment before surgery, unlike the prospective study that excludes patients who have received antibiotic treatment within 2 weeks prior to surgery. Therefore, confounding factors cannot be entirely excluded, potentially accounting for the different findings between the two studies.

Our study represented the first attempt to classify PJI patients based on BMI and assess the diagnostic value of traditional biomarkers, fibrinolytic biomarkers, and other inflammatory markers. However, it is crucial to acknowledge the limitations of this study. Firstly, it was a retrospective study conducted at a single center, which inevitably introduced inherent data biases. Secondly, the study focused on Chinese individuals, and racial differences may result in variations in the definition of “obesity.” Therefore, further validation of our research findings is necessary for other ethnic populations. Thirdly, the lack of a gold standard for PJI diagnosis introduces the possibility of misclassification of patients. Fourthly, the sample size in our study, particularly for PJI patients in the obese group, was limited. Thus, larger sample sizes and prospective studies are needed to ensure the reliability of our conclusions.

## Conclusions

In conclusion, among the biomarkers studied, CRP and CAR demonstrated the highest diagnostic value in the obese and non-obese groups, respectively. While the subgroup analysis did not yield optimal results, these findings do not diminish the promising potential of CRP and CAR as diagnostic biomarkers for PJI patients with varying BMI levels.

## Data Availability

The raw data supporting the conclusions of this article will be made available by the authors, without undue reservation.

## References

[B1] Boni L. Kuster S. P. Bartik B. Zbinden R. Zingg P. O. Achermann Y. (2018). Association of cutibacterium avidum colonization in the groin with obesity: A potential risk factor for hip periprosthetic joint infection. Clin. Infect. Dis. 67, 1878–1882. doi: 10.1093/cid/ciy379 29746626

[B2] Cohen E. Margalit I. Shochat T. Goldberg E. Krause I. (2021). Markers of chronic inflammation in overweight and obese individuals and the role of gender: A cross-sectional study of a large cohort. J. Inflammation Res. 14, 567–573. doi: 10.2147/JIR.S294368 PMC7920597 33658829

[B3] Font-Vizcarra L. Tornero E. Bori G. Bosch J. Mensa J. Soriano A. (2011). Relationship between intraoperative cultures during hip arthroplasty, obesity, and the risk of early prosthetic joint infection: a prospective study of 428 patients. Int. J. Artif. Organs. 34, 870–875. doi: 10.5301/ijao.5000026 22094568

[B4] Gundtoft P. H. Pedersen A. B. Varnum C. Overgaard S. (2017). Increased mortality after prosthetic joint infection in primary THA. Clin. Orthop. Relat. Res. 475, 2623–2631. doi: 10.1007/s11999-017-5289-6 28236084 PMC5638726

[B5] Houdek M. T. Wagner E. R. Watts C. D. Osmon D. R. Hanssen A. D. Lewallen D. G.. (2015). Morbid obesity: a significant risk factor for failure of two-stage revision total hip arthroplasty for infection. J. Bone Joint Surg. Am. 97, 326–332. doi: 10.2106/JBJS.N.00515 25695985

[B6] Issa K. Banerjee S. Kester M. A. Khanuja H. S. Delanois R. E. Mont M. A. (2014). The effect of timing of manipulation under anesthesia to improve range of motion and functional outcomes following total knee arthroplasty. J. Bone Joint Surg. Am. 96, 1349–1357. doi: 10.2106/JBJS.M.00899 25143495

[B7] Kapadia B. H. Berg R. A. Daley J. A. Fritz J. Bhave A. Mont M. A. (2016). Periprosthetic joint infection. Lancet 387, 386–394. doi: 10.1016/S0140-6736(14)61798-0 26135702

[B8] Koh C. K. Zeng I. Ravi S. Zhu M. Vince K. G. Young S. W. (2017). Periprosthetic joint infection is the main cause of failure for modern knee arthroplasty: an analysis of 11,134 knees. Clin. Orthop. Relat. Res. 475, 2194–2201. doi: 10.1007/s11999-017-5396-4 28573549 PMC5539036

[B9] Laaksonen D. E. Niskanen L. Nyyssonen K. Punnonen K. Tuomainen T. P. Valkonen V. P.. (2004). C-reactive protein and the development of the metabolic syndrome and diabetes in middle-aged men. Diabetologia 47, 1403–1410. doi: 10.1007/s00125-004-1472-x 15309290

[B10] Leff R. D. Akre S. P. (1986). Obesity and the erythrocyte sedimentation rate. Ann. Intern. Med. 105, 143. doi: 10.7326/0003-4819-105-1-143_2 3717798

[B11] Lowik C. Zijlstra W. P. Knobben B. Ploegmakers J. Dijkstra B. de Vries A. J.. (2019). Obese patients have higher rates of polymicrobial and Gram-negative early periprosthetic joint infections of the hip than non-obese patients. PloS One 14, e215035. doi: 10.1371/journal.pone.0215035 PMC6453483 30958847

[B12] Marongiu G. Contini A. Cozzi Lepri A. Donadu M. Verona M. Capone A. (2020). The treatment of acute diaphyseal long-bones fractures with orthobiologics and pharmacological interventions for bone healing enhancement: a systematic review of clinical evidence. Bioengineering (Basel) 7 (1), 22. doi: 10.3390/bioengineering7010022 32102398 PMC7148449

[B13] Matthews P. C. Berendt A. R. McNally M. A. Byren I. (2009). Diagnosis and management of prosthetic joint infection. BMJ 338, 0. doi: 10.1136/bmj.b1773 19482869

[B14] Nguyen X. M. Lane J. Smith B. R. Nguyen N. T. (2009). Changes in inflammatory biomarkers across weight classes in a representative US population: a link between obesity and inflammation. J. Gastrointest. Surg. 13, 1205–1212. doi: 10.1007/s11605-009-0904-9 19415399 PMC2693771

[B15] Parvizi J. Tan T. L. Goswami K. Higuera C. Della Valle C. Chen A. F. (2018). The 2018 definition of periprosthetic hip and knee infection: an evidence-based and validated criteria. J. Arthroplasty. 33, 0. doi: 10.1016/j.arth.2018.02.078 29551303

[B16] Pevzner L. Swank M. Krepel C. Wing D. A. Chan K. Edmiston C. J. (2011). Effects of maternal obesity on tissue concentrations of prophylactic cefazolin during cesarean delivery. Obstet. Gynecol. 117, 877–882. doi: 10.1097/AOG.0b013e31820b95e4 21422859

[B17] Probasco W. V. Cefalu C. J. Lee R. Lee D. Gu A. Dasa V. (2020). Prevalence of idiopathically elevated ESR and CRP in patients undergoing primary total knee arthroplasty as a function of body mass index. J. Clin. Orthop. Trauma 11, S722–S728. doi: 10.1016/j.jcot.2020.05.031 32999546 PMC7503783

[B18] Roschke E. Kluge T. Stallkamp F. Roth A. Zajonz D. Hoffmann K. T. (2021). Use of PET-CT in diagnostic workup of periprosthetic infection of hip and knee joints: significance in detecting additional infectious focus. Int. Orthop. 46, 0. doi: 10.1007/s00264-021-05218-8 PMC8840933 34618195

[B19] Roth A. Khlopas A. George J. Churchill J. L. Molloy R. Mont M. A.. (2019). The effect of body mass index on 30-day complications after revision total hip and knee arthroplasty. J. Arthroplasty. 34, S242–S248. doi: 10.1016/j.arth.2019.02.005 30846315

[B20] Shi W. Jiang Y. Tian H. Wang Y. Zhang Y. Yu T.. (2023). C-reactive protein-to-albumin ratio (CAR) and C-reactive protein-to-lymphocyte ratio (CLR) are valuable inflammatory biomarker combination for the accurate prediction of periprosthetic joint infection. Infect. Drug Resist. 16, 477–486. doi: 10.2147/IDR.S398958 36721632 PMC9884435

[B21] Shi W. Jiang Y. Wang Y. Zhang C. Yu T. Li T. (2022). The diagnostic value of various inflammatory biomarkers for diagnosing periprosthetic joint infection is gender-specific. J. Inflammation Res. 15, 3975–3982. doi: 10.2147/JIR.S364309 PMC9291658 35860231

[B22] Shi W. Wang Y. Zhao X. Yu T. Li T. (2021). CRP/albumin has a promising prospect as a new biomarker for the diagnosis of periprosthetic joint infection. Infect. Drug Resist. 14, 5145–5151. doi: 10.2147/IDR.S342652 34908848 PMC8664647

[B23] Visser M. Bouter L. M. McQuillan G. M. Wener M. H. Harris T. B. (1999). Elevated C-reactive protein levels in overweight and obese adults. JAMA 282, 2131–2135. doi: 10.1001/jama.282.22.2131 10591334

[B24] Wang L. Zhou B. Zhao Z. Yang L. Zhang M. Jiang Y.. (2021). Body-mass index and obesity in urban and rural China: findings from consecutive nationally representative surveys during 2004-18. Lancet 398, 53–63. doi: 10.1016/S0140-6736(21)00798-4 34217401 PMC7617101

[B25] Watts C. D. Wagner E. R. Houdek M. T. Lewallen D. G. Mabry T. M. (2015). Morbid obesity: increased risk of failure after aseptic revision TKA. Clin. Orthop. Relat. Res. 473, 2621–2627. doi: 10.1007/s11999-015-4283-0 25845948 PMC4488195

[B26] Whitehouse M. R. Parry M. C. Konan S. Duncan C. P. (2016). Deep infection after hip arthroplasty: staying current with change. Bone Joint J. 98-B, 27–30. doi: 10.1302/0301-620X.98B1.36294 26733637

